# Multidisciplinary and Tailored Treatment of Locally Advanced Breast Cancer in Progression during Neoadjuvant Chemotherapy: Case Report

**DOI:** 10.3390/curroncol31050217

**Published:** 2024-05-16

**Authors:** Letizia Cuniolo, Marco Gipponi, Federica Murelli, Francesca Depaoli, Chiara Cornacchia, Simonetta Franchelli, Marianna Pesce, Elena Ronda, Stefano Picardi, Raquel Diaz, Francesca Poggio, Daniele Friedman, Franco De Cian, Piero Fregatti

**Affiliations:** 1 Department of Surgical Sciences and Integrated Diagnostic (DISC), School of Medicine, University of Genoa, 16132 Genoa, Italy; 2 Breast Surgery Unit, IRCCS Ospedale Policlinico San Martino, 16132 Genoa, Italy; marco.gipponi@hsanmartino.it (M.G.);; 3 Department of Medical Oncology, U.O. Oncologia Medica 2, IRCCS Ospedale Policlinico San Martino, 16132 Genoa, Italy

**Keywords:** locally advanced breast cancer (LABC), progressive disease (PD), salvage treatment (ST)

## Abstract

Locally advanced breast cancer (LABC) is a complex disease that requires a multidisciplinary approach. Neoadjuvant chemotherapy (NAC) is usually performed in order to achieve loco-regional radical resection; although its importance in the multidisciplinary approach to LABC is well recognized, a small number of patients show Progressive Disease (PD). No standard salvage treatment (ST) has been defined and different strategies can be adopted, such as second-line systemic therapies, radiation therapy, and surgery. Herein, a case of LABC in PD during NAC is reported with a literature review, with the aim of highlighting the importance of a tailored multidisciplinary treatment for each patient.

## 1. Introduction

Locally advanced breast cancer (LABC) is an extensive breast cancer, without distant metastases, which may be resectable or unresectable. As compared to early breast cancer, it bears a worse prognosis due to unfavorable biological factors and the wider extent of loco-regional disease. Hence, a multidisciplinary approach is essential for the management of this challenging disease.

Because LABC patients have a high risk of metastatic disease, a complete staging workup including a physical examination, blood tests, and imaging of the chest, abdomen, and bone before the initiation of any treatment is needed [1,2].

Systemic therapy with anthracyclines and taxanes should be the initial treatment [1]. In HR (Hormone Receptor)-positive LABC, endocrine therapy must be considered. In c-erb-2-positive LABC, concurrent taxane and anti-HER2 therapy is recommended, and anthracycline should be administered sequentially with the anti-HER2 therapy [1].

Following neoadjuvant chemotherapy (NAC), with or without neoadjuvant radiation therapy (NART), surgery will be possible in many patients and consists of a mastectomy with axillary dissection in most cases [1].

Despite the importance of NAC, in some cases, tumor progression during therapy may occur. The main causes are represented by drug resistance, cancer heterogeneity, the tumor microenvironment, and the evolution of the neoplasm’s receptor status [3,4,5]. At present, there is no standard salvage treatment (ST) to implement in the case of disease progression during NAC. The type of ST is discussed by a multidisciplinary team, which includes medical and radiation oncologists and surgeons. Various strategies can be considered, such as a change in systemic therapy, radiotherapy (alone or in addition to chemotherapy), and surgery [6].

Herein, the clinical history of a 55-year-old post-menopausal woman with LABC who developed local Progressive Disease (PD) during NAC is reported. She was treated at San Martino Polyclinic Hospital in Genoa, in our breast unit. In this case report, we aimed to emphasize the importance of a salvage treatment specifically tailored for this patient with LABC in progression during NAC in order to improve local and distant disease control.

## 2. Case Presentation

The patient detected a lump in the upper outer quadrant of the right breast in September 2021 and she was visited at another hospital. Clinically, she had a palpable tumor in the right upper quadrant with skin redness; moreover, multiple lymph nodes were detectable in the homolateral axilla, the largest being about 15 mm. The mammography and breast Ultrasound (US) showed a suspicious neoplasm of 37 × 17 mm, and the needle-biopsy demonstrated a high-grade (G3), estrogen receptor (ER)-positive (40%), progesterone receptor (PgR)-negative, Ki67 = 40%, c-erb-2 protein-positive (3+) invasive ductal breast cancer. The bone scintigraphy and total-body Computed Tomography (CT) scan were both negative for distant metastases. After a multidisciplinary team (MDT) evaluation, NAC was given, including 4 cycles of Epirubicin-Cyclophosphamide (EC) followed by Trastuzumab and Paclitaxel for 12 weeks. The genetic assessment for the BRCA-1 and BRCA-2 mutations was negative.

After 4 cycles of EC, the breast US showed stable disease; moreover, although the previous clinically positive axillary lymph node of about 15 mm was no longer visible, a new axillary lymph node 5 mm in diameter was highlighted, while the remaining lymph nodes had a reactive appearance. After 6 cycles of NAC, a slight dimensional reduction in the breast lump (27 × 10 mm vs. 37 × 17 mm) was observed at the breast US, and the pathologic lymph nodes into the axilla were no longer detectable; therefore, the patient continued the ongoing NAC regimen.

**Figure 1:**
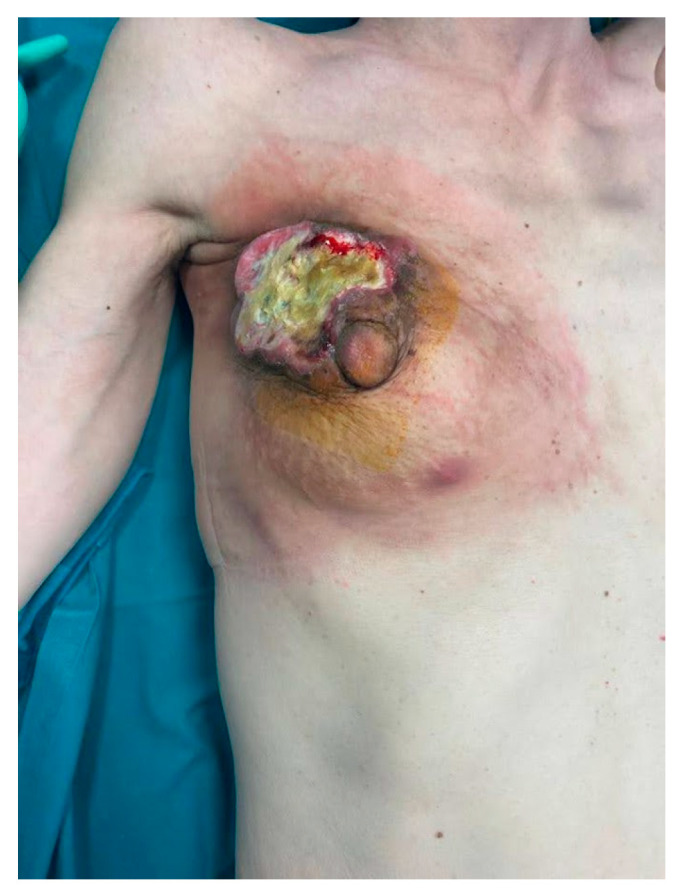
After NAC and NART and before surgery the tumor was locally advanced, with skin ulceration and necrosis.

After five months of NAC, breast Magnetic Resonance (MR) showed a minimal partial response of the breast lesion (25 × 10 mm in diameter) with negative axilla. However, the skin redness and retraction had increased, with worsening ulceration (Figure 1). A second US-guided biopsy was then suggested, and G3, ER-negative, PgR-negative, Ki67 = 75%, c-erb-2-positive (3+) invasive ductal carcinoma was confirmed. After a new MDT discussion, the breast disease was deemed unresectable, and Trastuzumab Deruxtecan in the expanded access program (EAP) was started. However, after 4 cycles, due to unsatisfactory local control of the disease, radiation therapy (RT) of the breast with concurrent chemotherapy (Trastuzumab, Tucatinib, and Capecitabine) was given.

After three months of the new NAC regimen, a breast US showed a further increase in the primary neoplasm (48 × 37 mm vs. 48 × 18 mm) with skin invasion, and at the CT scan, no clear cleavage plane of the breast lesion from the pectoralis major muscle was appreciable. Hence, the ongoing NAC regimen was stopped, and it was replaced with Carboplatin every 3 weeks. The breast MR re-evaluation confirmed a clear-cut local PD with a breast mass measuring 64 × 55 × 49 mm with concomitant chest wall infiltration, as indicated by the chest CT scan, which highlighted tumor invasion of the skin plane, pectoralis major and minor muscles, and intercostal muscles at the 3rd and 4th intercostal spaces; an homolateral axillary lymph node (8 × 6 mm) was also appreciable.

At this point, in April 2023, the patient came to the attention of our breast unit. After the breast and thoracic surgeon’s assessment, the lesion was deemed resectable, though with a high risk of 3rd rib resection.

Preoperatively, the ulcerated skin of the right breast was sampled for a cultural examination with isolation of a multisensitive *Acinetobacter Baumanni*; hence, Amoxicillin/Clavulanate 1 g × 3/day and Metronidazole 500 mg × 3/day antibiotic therapy was given.

**Figure 2:**
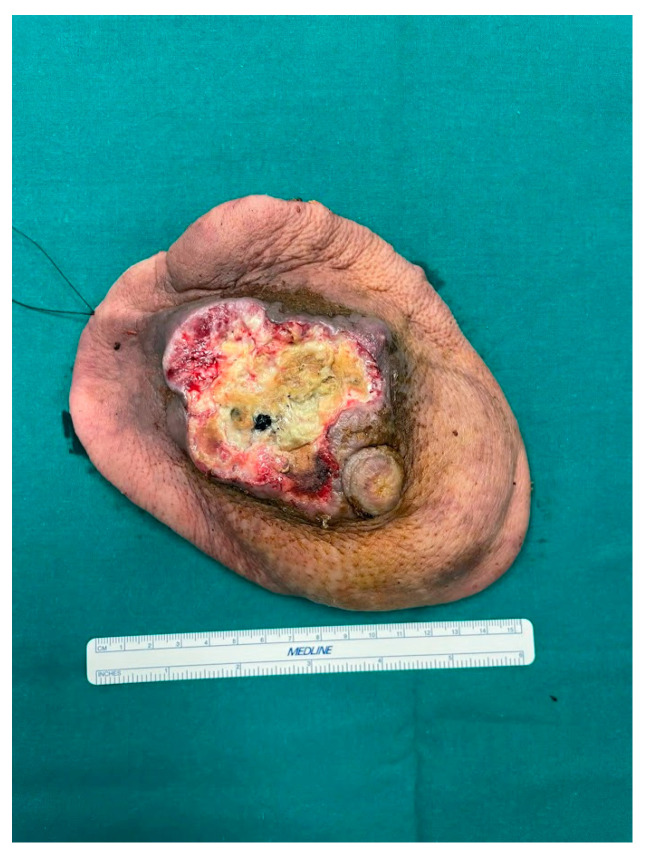
The specimen measured 17.5 × 12 × 5 cm.

**Figure 3:**
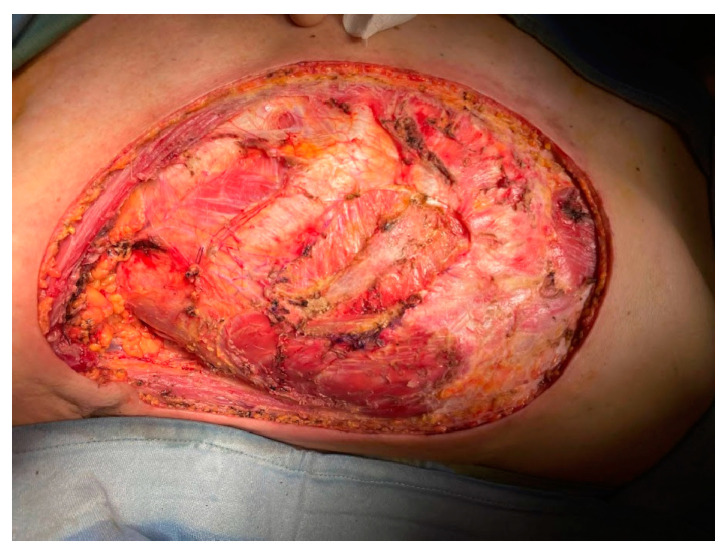
Local state after Halsted mastectomy.

In May 2023, the patient underwent a Halsted radical mastectomy with partial periosteal resection of the 4th rib and partial resection of the 3rd and 4th external intercostal muscles (Figure 2 and Figure 3); intraoperative frozen sections were performed and were negative for deep infiltration. However, a precautionary enlargement on the medial, lateral, and deep sides of the 4th external intercostal muscle was performed for definitive histology. Intraoperative cultural samples were also undertaken, and again, a multisensitive *Acinetobacter Baumannii* was isolated, so the ongoing antibiotic therapy with Amoxicillin/Clavulanate was replaced by a targeted antibiotic therapy with Meropenem 1 g × 4/day.

**Figure 4:**
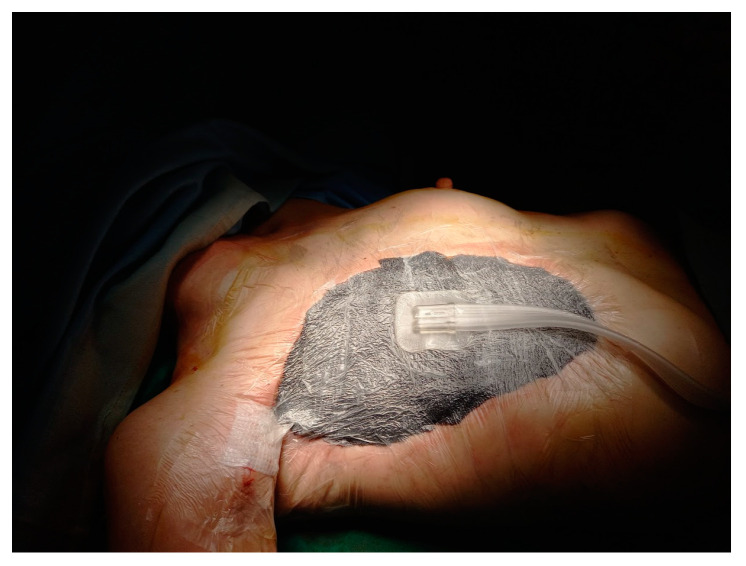
Local state after application of VAC therapy.

**Figure 5:**
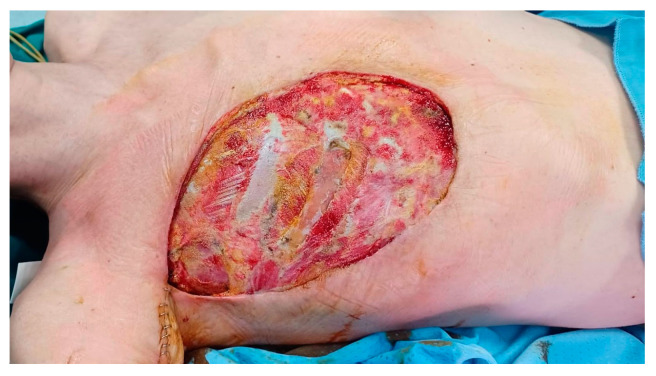
Re-epithelialization of wound margins after 20 days of VAC therapy.

Definitive reconstruction was postponed until the findings of the cultural examination and definitive histology were available. Moreover, the patient’s poor general conditions (BMI = 15.6, albumin 4.1 g/dL, and hemoglobin 9.4 g/dL) did not allow for a flap reconstruction. In the meantime, negative-pressure Vacuum-Assisted Closure (VAC) therapy of 100 mmHg was applied, with a subsequent reduction at 80 mmHg due to the patient’s poor tolerance (Figure 4). It was maintained for a period of 20 days (Figure 5), during which it was replaced three times, with serial sampling for cultural examination, the last being positive for multiresistant *Staphylococcus epidermidis* that was treated with Linezolid 600 mg × 2/day in addition to Meropenem.

At definitive histology, a G3, ER-negative, PgR-negative, *Ki67* = 90%, c-erb-2 negative invasive ductal carcinoma was diagnosed with free tumor margins; each specimen of the medial, lateral, and deep edges of the 4th external intercostal muscle was free from neoplastic infiltration, as well. Nine lymph nodes were isolated, each of them being negative for the presence of metastases (ypT4b ypN0 Mx, UICC-TNM 2018). The expression of PD-L1 was also evaluated, which was negative. Therefore, the patient did not have access to immunotherapy.

**Figure 6:**
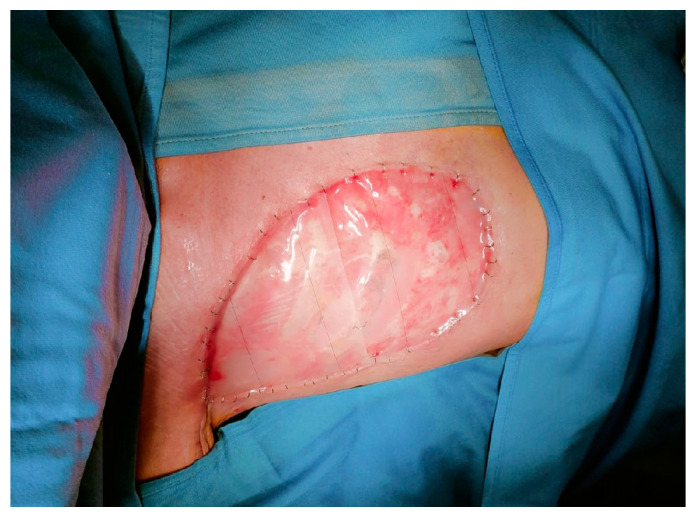
Application of Integra^®^ matrix.

After VAC therapy, a bioengineered dermal matrix (Integra^®^, Integra Lifescience, Princeton, NJ, USA) was placed into the surgical bed (Figure 6). The patient was discharged on the 6th post-operative day, and oral Linezolid 600 mg × 2/day was given until the 10th post-operative day. The dressing was replaced twice a week for a month in the outpatient clinic; hence, the patient underwent a new reconstructive procedure by means of a full-thickness graft taken from the suprapubic region with an abdominoplasty-type design. In the following days, greenish wound secretions were observed, and an infection by *Pseudomonas Aeruginosa* and *Enterobacter Cloacae Complex* was diagnosed; intravenous antibiotic therapy was immediately started, giving Piperacillin/Tazobactam 4.5 g × 4/day as the empiric antibiotic therapy at first, followed by Ciprofloxacin 750 mg × 2/day and, finally, Ceftolozane/Tazobactam 1.5 g × 3/day.

The applied graft was successful on approximately 40% of the treated surface. There was partial exposure of the 3rd and 4th ribs due to an associated osteomyelitis caused by a multisensitive *Pseudomonas Aeruginosa*; antibiotic therapy with Ceftolozane/Tazobactam was suspended and it was replaced with intravenous Meropenem 1 g × 3/day.

Subsequently, the patient’s general conditions and BMI improved due to a better nutritional intake, so the reconstructive surgery of the chest wall was completed by means of a vertical rectus abdominis pedicled myocutaneous flap (VRAM) to cover the exposed ribs. A local rotation flap was used in the lateral thoracic area to close the infero-lateral loss of substance, and a full-thickness dermo-epidermal graft was taken from the medial surface of the right arm to cover a portion of the aforementioned myocutaneous flap. During the surgical intervention, a sample of periosteal tissue was taken for microbiological examination, which again tested positive for multisensitive *Pseudomonas Aeruginosa*. The Infectious Disease Team suggested an antibiotic therapy with Ceftolozane/Tazobactam 1.5 g × 3/day and Amikacin 900 mg/day. The post-operative course was uneventful, with an improvement in the local conditions and a reduction in all inflammatory acute phase proteins. The patient was discharged with intravenous antibiotic therapy at home by means of an elastomeric pump for a total of four weeks of therapy.

**Figure 7:**
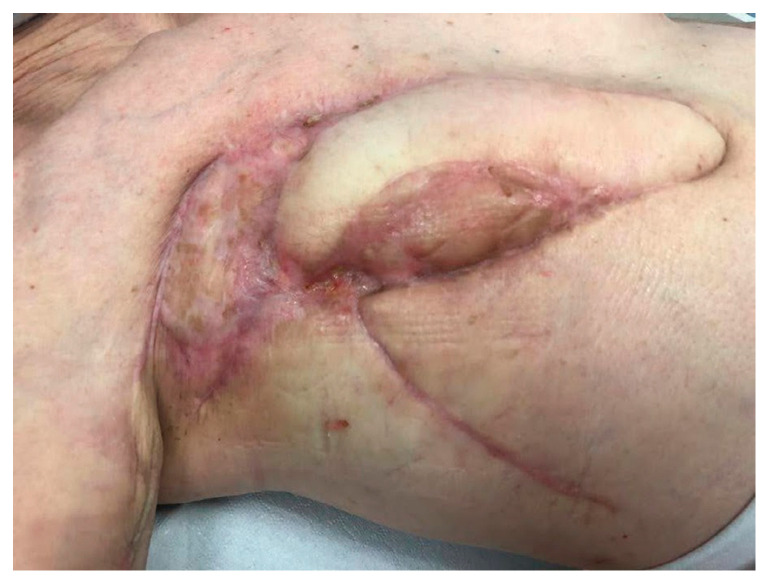
Complete wound healing was achieved 60 days after the last surgical procedure (VRAM, local rotation flap, and a full-thickness dermo-epidermal graft taken from the medial surface of the right arm).

Clinically, she had a progressive improvement at the local site with complete healing being achieved 60 days after the last surgical procedure (Figure 7). Finally, the patient was in good general condition with no evidence of PD after total-body CT scans, so no adjuvant post-operative CT was given, and she underwent close follow-up with negative findings until now (eight months follow-up).

**Figure 8:**
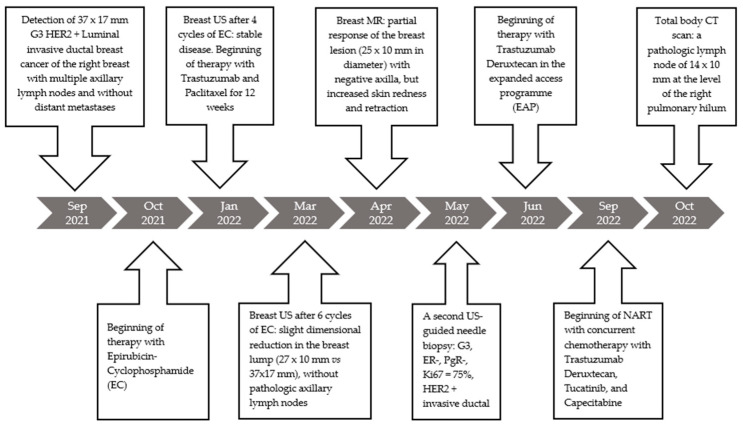

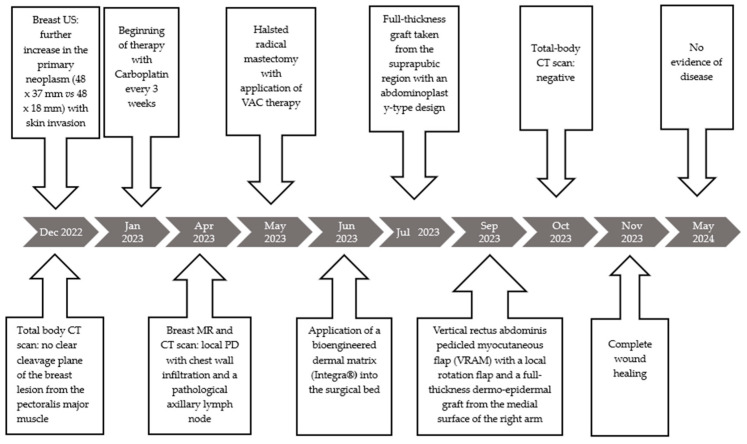
All the important examinations, diagnoses, and therapies are listed in this timeline.

All the important examinations, diagnoses, and therapies are summarized in a timeline (Figure 8).

## 3. Discussion

LABC is a complex disease requiring a multidisciplinary approach. A personalized therapeutic approach is mandatory according to tumor biological features, menopausal status, and co-morbidity factors. NAC is usually performed for achieving loco-regional radical resection as well as to reduce the extent of surgery [7,8]. The use of anthracycline-based and taxane-based drugs is associated with a better chance of obtaining a clinical and a pathological response [9]. The addition of neoadjuvant Trastuzumab to NAC improves the event-free survival, survival, and tumor response in patients with c-erb-2-positive LABC [10].

The main studies about LABC and their results are presented in Table 1.

Although the importance of NAC in the multidisciplinary approach to LABC is well recognized, a small number of patients experience only a less than optimal response or even show PD, as in our case, so that breast-conserving surgery or even mastectomy may be impossible [11]. PD during NAC for breast cancer is defined as an increase in the tumor size or appearance of new tumor lesions in the breast, lymph nodes, or distant organs [12]. Some trials reported that the rate of PD during NAC is about 3–5% [6,13]. Caudle et al. found that predictors of PD included African American race, a large initial tumor size, a negative estrogen and progesterone receptor status, high Ki-67 scores, and a high nuclear grade [13].

Drug resistance is one of the main causes of therapeutic failure. The best-known drug resistance mechanisms include expression and activity of drug-metabolizing enzymes, increased expression levels or enhanced functions of drug efflux systems, overexpression of the Glutathione Detoxification System, the DNA damage repair mechanism, inhibition of cell apoptosis and autophagy, epithelial-to-mesenchymal transition, the activity of exosomes, and the tumor microenvironment. Therefore, one of the more commonly used strategies to overcome drug resistance is to combine multiple chemotherapy drugs [14].

Another cause of therapeutic failure is the change in HR status during PD, as presented in our case report. This phenomenon could be the result of genetic and epigenetic mechanisms, intratumor heterogeneity, the tumor microenvironment, and the selective pressure of anticancer treatments [15]. Indeed, cytotoxic therapies have been shown to modify HR expression in BC cells [16]. Of note, intratumor heterogeneity of HR expression has been associated with a poorer prognosis due to the higher number of biologically different tumor clones with an increased ability to adapt to adverse growth conditions, including the selective pressure of cytotoxic agents [17]. Therefore, a biological tumor re-assessment after NAC in PD is mandatory in order to tailor the following treatment [15].

Whenever PD does occur during NAC treatment, no standard ST has been defined and different options may be pursued, such as the change in systemic therapies, radiation therapy (alone or in addition to chemotherapy), and surgery [6]. The type of ST is decided by a multidisciplinary team, including a medical and radiation oncologist and surgeon. One of the options to face PD during NAC is to switch systemic therapy to a different regimen than the initially planned treatment. Another salvage strategy is the use of NART, with or without NAC, which has shown in some trials satisfactory results regarding loco-regional control, survival, and post-surgical outcomes [18,19]. Finally, surgery is a feasible approach in patients thought to be operable at the time of PD or in patients treated with non-surgical ST who became operable thereafter. Raphael J et al. found that a significant number of non-operable patients who initially progressed on NAC (69%) were able to undergo surgery after receiving different types of non-surgical ST [6]. In their trial, upfront surgery, NART alone, and NART associated with NAC have been used as ST with approximately the same frequency for patients with hormone-sensitive PD, while upfront surgery has been employed as the ST in the 72% of the patients with c-erb-2 PD and NART in association with NAC administered as the ST in the 53% of the patients who presented a triple-negative PD [6]. A mastectomy with axillary dissection represents the standard technique for patients with LABC not eligible for breast-conserving surgery after NAC [20]. In fact, the loco-regional relapse rate has been shown to be higher after conservative local surgery than after radical surgery [21].

No difference in survival was already found between all the ST regimens, even if some clinical results have been promising, providing a starting point for new research [6].

In our case, upfront surgery was not a feasible option at the time of PD; therefore, first a systemic therapy switch and then an association of NAC and NART were employed. These STs have made demolitive but radical surgery possible. Collaboration with thoracic surgeons allowed us to be prepared for a possible resection of part of the chest wall and an eventual repair of substance loss by various reconstructive techniques. The latter procedure was not necessary in view of the absence of disease in the samples of the periosteum and intercostal muscles sent for intraoperative frozen section examination.

Following surgical resection of LABC when local PD does occur, as in our case, extensive and complex wounds may require special care because prolonged wound healing can delay adjuvant CT and RT. The achievement of complete wound healing by means of VAC therapy is known to be effective in the management of different complex wounds. It has been shown to improve wound healing by removing excessive interstitial fluid, increasing tissue blood flow, stimulating granulation tissue and neo-vascularization, and decreasing bacterial colonization of wounds, as confirmed in randomized clinical trials [22,23,24,25,26].

The response to NAT in breast cancer is considered a crucial indicator of the tumor’s sensitivity to the therapy itself. It is widely accepted that a complete or partial response to treatment is associated with a better prognosis and increased Disease-Free Survival (DFS), Distant Disease-Free Survival (DDFS), and Overall Survival (OS) in breast cancer patients. Thus, patients achieving a complete pathological response to NAT have a significantly better prognosis compared to those who do not achieve this goal [27,28,29]. Therefore, evaluating the response to NAT is essential in clinical decision-making and guiding follow-up and subsequent therapies [30,31,32].

**Table 1:** The table summarizes the main studies concerning LABC and their findings.

Authors and References	Title of Paper	Study Design	Main Findings
Whitman GJ, Strom EA [2]	Workup and Staging of Locally Advanced Breast Cancer	Review	In patient with LABC, accurate workup and staging evaluations are extremely important in facilitating appropriate multidisciplinary treatment.
Petrelli F, Coinu A, Lonati V et al. [9]	Neoadjuvant dose-dense chemotherapy for locally advanced breast cancer: a meta-analysis of published studies	Meta-analysis	Dose-dense NAC, despite not leading to a significant increase in survival, increases the possibility of achieving a pathologic complete response (pCR) in operable and LABC by 46.7%.
Gianni L, Eiermann W, Semiglazov V et al. [10]	Neoadjuvant chemotherapy with trastuzumab followed by adjuvant trastuzumab versus neoadjuvant chemotherapy alone, in patients with HER2-positive locally advanced breast cancer (the NOAH trial): a randomised controlled superiority trial with a parallel HER2-negative cohort	Randomized controlled superiority trial	Trastuzumab significantly improved event-free survival in patients with HER2-positive breast cancer.
Raphael J, Paramsothy T, Li N, Lee J, Gandhi S [13]	A single-institution experience of salvage therapy for patients with early and locally advanced breast cancer who progress during neoadjuvant chemotherapy	Retrospective cohort study	Patients progressing on NAT responded well to ST, became operable, and had promising survival outcomes. Appropriate selection of ST is crucial.
Tryfonidis K, Senkus E, Cardoso MJ, Cardoso F [20]	Management of locally advanced breast cancer—perspectives and future directions	Review	Optimal management of LABC requires a multidisciplinary approach, a well-coordinated treatment schedule, and close cooperation between medical, surgical, and radiation oncologists.
Touboul E, Buffat L, Lefranc JP et al. [21]	Possibility of conservative local treatment after combined chemotherapy and preoperative irradiation for locally advanced noninflammatory breast cancer	Prospective study	Induction chemotherapy followed by preoperative irradiation may permit the selection of some patients with locally advanced breast cancer for conservative treatment. However, the impact of this treatment modality on long-term survival remains to be established.
Roininen N, Haapasaari KM Karihtala P [33]	The role of redox-regulating enzymes in inoperable breast cancers treated with neoadjuvant chemotherapy	Retrospective observational study	Redox-regulating enzymes may serve as potential prognostic factors in primarily inoperable breast cancer patients.

In this case report, we aimed to highlight that, thanks to the multidisciplinary treatment undertaken, the patient is disease-free, despite disease progression during NAT representing a negative prognostic factor that threatens the patient’s survival. We are aware that a longer follow-up period is necessary to obtain more meaningful insights. However, considering the current condition of the patient, who remains disease-free after several months of follow-up, we are confident in the success of the multidisciplinary and tailored treatment undertaken.

The strengths of our work were the multidisciplinary management of the case, which allowed for combining several strategies of ST, and the variety of reconstructive techniques employed accordingly to highly destructive surgery. Moreover, the collaboration with infectious diseases specialists, pain therapists, physiotherapists, and nutritionists allowed for managing in their totality the collateral problems to the patient’s main disease. However, as a case report, this article has obvious limitations, such as its retrospective design and the impossibility to generalize and to establish a cause–effect relationship.

## 4. Conclusions

LABC is typically an aggressive disease due to its adverse biological features and advanced stage at presentation, with an overall poor prognosis [27]. In particular, PD during NAT represents a negative prognostic factor, with a significant impact on the patient’s survival. This case report emphasizes the importance of a multidisciplinary, tailored treatment approach for each patient with LABC, especially when disease progression occurs during NAC. The combination of different medical, radiation, and surgical therapeutic options is crucial to improving loco-regional and systemic disease control.

## Data Availability

The data presented in this study are available in this article.
